# Supplementary material for: Are carbon emissions trading and green financial instruments synergistic? -Comprehensive quantitative research based on content analysis
Source: PLoS One. 2024 Mar 7;19(3):e0298601. doi: 10.1371/journal.pone.0298601 (PMC10919601; doi:10.1371/journal.pone.0298601)
Supplement: S1 Data — (ZIP) [file pone.0298601.s001.zip › Data/地级市Z_F工作报告.zip!/╣π╢1⁄2/╟σ╘╢╩╨/└┤╫╘╣1⁄2╓┌║┼í╛┬φ┐╦╩2╛▌═°í┐_2015.docx]

各位代表：

我代表市人民政府，向大会报告政府工作，请予审议。并请政协各位委员和其他列席人员提出意见。

2014年工作回顾

刚刚过去的2014年，是本届政府任内承上启下的一年。在市委的正确领导下，在市人大、市政协的监督支持下，我们认真落实市六届人大四次会议和市人大常委会确定的各项工作，突出发展、改革、稳定、民生主题，扎实做好政府工作，实现了经济社会发展新的进步。

经济发展实现了稳中提质。我们主动适应经济发展新常态，坚持稳中求进总基调，积极应对经济下行压力，大力推进改革创新，保持了经济企稳回升的良好态势。全市完成生产总值1187.7亿元，同比增长7.94%；来源于清远的财政总收入278亿元，增长12.8%；全市一般公共预算收入102.6亿元，增长10.6%；固定资产投资增长22.6%，其中工业投资增长34.6%；社会消费品零售总额增长11.5%；城乡居民人均可支配收入增长9.9%。产业结构进一步优化，区域发展差距缩小，经济质量效益提升，转型发展取得初步成效。

发展条件有了更大改善。我们积极推进交通、园区和“一心两核”﹝1﹞城市建设，乐广、二广高速建成通车，一批国省道完成改造。燕湖新城首轮征地拆迁基本完成，骨架路网和起步区建设有序铺开。园区配套进一步完善，产业承接能力有效增强。在粤东西北率先出台权责清单﹝2﹞、后续监管清单和企业投资负面清单，营商环境得到优化。广清一体化进程加快，清远获得了更多的发展机会。

民生保障切实得到加强。我们投入更多财力保障基本民生，全年民生领域投入资金155.7亿元，增长19.2%，占财政总支出的73%。底线民生保障水平大幅提高，教育、医疗等公共服务均等化水平进一步提升，十件民生实事基本完成，北江流域保护治理和大气污染防治成果显现。

过去一年，我们主要抓了以下八项工作：

一、力促经济实现更有质量的增长

着力破解项目落地难问题。采取“先安置、后拆迁”的模式，妥善解决失地农民生活安置和收入来源问题，顺利完成了大批重点项目的征地拆迁工作。及时调整土地利用计划，保障了重点项目用地需求。推行建设项目“集装箱”并联审批﹝3﹞，开展重点项目“直接落地”﹝4﹞试点。落实领导包干机制，强化重点项目建设考核。省、市重点项目共完成投资362.4亿元，占年度投资计划的103.1%，完成率比上年提高了6.9个百分点。其中，省重点项目完成年度投资计划的120%，完成率上升到全省第9位。金发科技﹝5﹞、长隆森林乐园﹝6﹞、天安智谷﹝7﹞等一批重大产业项目顺利开工。

围绕重点区域和重点产业招商。把广佛地区作为重点招商区域，把制造业和现代服务业作为重点引进产业，加强与商会和行业协会的对接，实行市、县、园区联动招商。全市新引进产业项目177个，计划投资366亿元，累计实际投入57.5亿元。欧派家居﹝8﹞、博纳医疗器械﹝9﹞等一批优质项目落户。5家银行﹝10﹞在清远设立分支机构。

推动现有企业增资扩产。帮助有增资扩产意愿的企业解决用地问题，落实技改扶持政策，实施“百家企业帮扶”工程；召开7场政企沟通联席会议，帮助企业解决173件困难事项；落实结构性减税政策，共为企业减负8.4亿元；支持企业到“新三板”﹝11﹞挂牌，鼓励规下企业转为规上企业。全市49家重点扶持企业增资78.7亿元，34家企业完成技改投资15.3亿元。新增工业投资135.5亿元；新增规上工业企业15家，新增产值165亿元；新增国家4A级旅游景区4个。

加强产学研对接合作。“华炬科技企业孵化器”﹝12﹞等公共平台建设加快推进，清远高新区升级国家级高新区获省批准。全市新增高新技术企业6家，参与制（修）订国家标准3项、省地方标准4项，首次获得中国专利优秀奖、省科技进步一等奖，首次承担国家863计划﹝13﹞项目任务。140家企业与88家高校、科研院所建立了产学研合作关系。

二、改革制约发展的关键环节

在粤东西北率先出台“三份清单”。颁布了商事改革后续监管清单，明确了41个部门211项监管事项；向社会公布了企业投资负面清单，涉及17个行业1145个项目，成为全省第二个出台投资负面清单的地级市；确定了市级政府部门首批权责清单，涉及7个部门1154个事项。完成了市、县两级政府机构改革；制定了第三批行政审批制度改革事项目录，调整审批事项330项，调整比率达50.6%；将37项企业投资项目技术性审查推向市场；市级36个部门行政审批实现“两集中、三到位”﹝14﹞，进驻审批业务684项。深化商事制度改革，前置审批事项减少近9成，新登记各类市场主体同比增长14.3%，其中新增企业主体增长50.1%；探索推进电商企业集群注册，全市新增电商企业105家。深化学校、医院去行政化改革，在11家单位试点法人治理结构。

推进涉农资源和平台整合。推进农村土地资源、财政涉农资金和涉农服务平台整合，共整合财政涉农资金1.2亿元、土地资源31.8万亩。积极实施农村普惠金融“八项行动”﹝15﹞，建成682个金融服务站，“政银保”﹝16﹞合作担保基金实现县域全覆盖。三个试点镇村委会下沉设置顺利推进，土地承包经营权确权试点铺开。我市成为国家农村改革试验区，中央农办农村改革试验联系点，省创新农村基层社会治理、深化农村综合改革试点市。阳山县建成县镇村三级联动的产权交易服务网络。

启动城市社区治理改革试点。在清城、英德等4个县（市、区）开展城市社区治理改革试点，实施23个社会创新项目和52个平安清远创建工程项目。市本级新增27家枢纽型社会组织，行业协会（商会）与政府部门全面脱钩。网上办事大厅覆盖全部镇街和758个村居，市、县两级进驻服务事项6349项。“两建”﹝17﹞平台、12345热线正式上线运行。

三、加快重大基础设施建设

努力实现“县县通高速”。连山县结束无高速公路历史，英德市区连通高速公路，各县（市、区）均实现高速公路通达。佛清从高速北段顺利更换项目业主，广清高速与清连高速连接线、汕昆高速清远段、汕湛高速清远至云浮段先行工程开工建设，汕湛高速惠州至清远段已确认为经营性项目，年内可望开工建设。利用社会资金启动了33个总里程达633公里的国省道改造项目，完成G106佛冈南段、广清大道、连怡公路、英坑公路等10条129公里改造任务。建设全市性治超监控网络，实施24小时治超执法。广清城轨一期工程清远段累计完成工程量的39.4%。北江千吨级航道整治先行工程正式动工。全市交通基础设施建设完成投资105亿元。

加快完善园区配套。一批园区骨干路网及供水、供电、通讯、污水处理等配套设施顺利推进，顺德清远（英德）经济合作区“两纵两横”路网框架初步形成，500千伏清远蓄能送出线路、220千伏石潭输变电等电网项目顺利投产。5个重点园区新引进项目42个，在建项目92个，实现工业增加值95亿元，同比增长39.7%。产业园区获得省扩能增效资金3.4亿元。

抓好信息基础设施建设。实施免费无线网络惠民工程，开放公共建筑支持无线基站建设，实现全市3G网络全覆盖，市县镇中心区域4G网络全覆盖，市中心区域公共场所无线网络免费服务。实施宽带提速工程，全市光纤入户累计达16.7万户。完成市、县两级应急指挥平台建设，城管、公安、交通等现有视频监控资源得到整合。推进政务信息资源库建设，归集政务信息资源215万条，大数据﹝18﹞建设开始起步。

四、大力改善城市功能环境

加快新区路网和安置区建设。燕湖新城完成详规和专项规划13项，完成征地1.2万多亩，启动76项道路和房建工程，累计完成投资71.4亿元。市中心区民安路、峡山西路、站前东路等24项工程基本完工；北江东路、人民东路、凤翔北路、清晖北路、黄腾峡大道等城市骨架路网进展顺利，可望年内完成；凤凰安置区等3个村民安置区全面动工；北江五桥完成投资1.5亿元，北江四桥动工建设。英德东岸新城完成征地1500多亩，完成投资1.5亿元，杨万里大道、滨江东路等项目加快建设。连州城西新区完成控制性详细规划编制，兴连大道绿化亮化、南门大道西等工程已基本完成。

推进建成区改造提升。以“五城同创”﹝19﹞为抓手，进一步提升中心城区环境功能，修复改造一批市政道路，整治交通黑点30个；8座生活垃圾中转站和旧城地下排污管网建设加快推进，飞来湖截污工程和周边路网建设全面完成；新增、优化公交线路10条，新投放公交车79辆；江南水厂一期、太和水厂扩建工程顺利启动；拆除澜水豪庭等一批违法建筑。各县城完成一批市政基础设施和道路改造提升工程，公共照明领域路灯节能改造全部完成。全市建成公共停车场12个，升级改造肉菜市场54个，建设绿道86公里。获得全国文明城市提名资格。

抓好镇级规划修编。以土地规划中期评估为契机，做好土规调整和中心镇规划修编工作。39个非县城建制镇已编制或正在编制总体规划，21个中心镇完成总规编制，13个镇（街）完成控规编制。汤塘温泉旅游小镇等特色小镇铺开建设。

五、全面对接广州的对口帮扶

建立完善帮扶机制。成立两市对口帮扶工作领导小组和对口帮扶指挥部，建立了区县结对帮扶机制，共召开7次两市党政联席会议。广州市、区两级共选派179名干部和专业技术人员到清远开展帮扶工作。20亿元广清投资基金已设立，10亿元广清产业园投资开发公司注册资金、8000万元区县帮扶资金全部到位。

推进以产业为重点的全面帮扶。广清产业园的园区道路、企业服务中心、污水处理厂、市政管网等项目开工建设，市级权限全面承接；园区签约项目20多个，计划投资100多亿元。各结对区县合作共建产业园区抓紧推进，签约引进产业项目100多个，计划投资500多亿元。启动非产业类帮扶事项120项，112所学校、10家县级以上医院与广州结成“一对一”帮扶关系，广铁一中、华师附小等一批优质学校的分校落户清远，广东药学院附属第一医院正式托管连南县人民医院；借助广州批发市场等平台，推广销售清远农产品；合办多场清远旅游推介活动；实现两地养老保险和失业保险关系无障碍转移接续。

力推两地交通和要素市场一体化。编制广清交通一体化规划，以打通“断头路”为重点，推进广州北与清远南的路网衔接。协调推进广清重要交通通道征地拆迁，共同推动广清城轨二期前期工作。广州产权交易所、广州股权交易中心在清远设立分支机构，发布交易项目600多个，涉及金额约50亿元，两地市场要素流动加快。

六、改善农村生产生活条件

搭建农村电商平台。与阿里巴巴合作建设全国首个农村电子商务市，4县（市、区）纳入首批试点，阳山成为全省首个试点县，华南首家“京东帮”﹝20﹞落户连州。全市建成3个县级农业电商平台、82个农村淘宝村级服务站。清远农村电商基地被列为2014年广东省电商峰会推广项目。天猫旗舰店﹝21﹞“双十一”当天销售清远鸡857万只。

发展现代农业。制定“清远桂花鱼”、“清远黑山羊”产品标准，启动农产品二维码溯源系统﹝22﹞。全市新增农民专业合作社﹝23﹞623家、国家地理标志保护产品2项，入选省十大名牌系列农产品3个。阳山县建成全省第一个种植类国家级供港澳蔬菜质量安全示范区。

实施中小河流整治。完成203公里中小河流“三清理”﹝24﹞工作，完成10宗92公里中央项目的治理。启动省级水利建设示范县项目247宗，完成投资3.7亿元。投入4.5亿元推进高标准基本农田建设。修复水毁水利工程3129宗。阳山白莲片区洪涝综合整治工程全面动工。完成253条美丽乡村规划，建成76条美丽乡村。完成733个低压电台区和460项农村电网升级改造任务。

铺开国家扶贫改革试验区建设。在81个重点帮扶村开展扶贫改革示范村创建工作，全市共落实帮扶资金14.7亿元，村均投入509万元；开展村帮扶项目6276个，户帮扶项目6.9万个；村集体经济收入平均达到7.9万元，贫困户人均收入增至6493元。完成农村低收入困难户住房改造6343户。

七、下大力气治理环境修复生态

启动北江流域保护治理工作。出台北江流域水质保护和治理工作实施方案，重点治理生活污水、农业面源、游船、工业、矿山开采等五个领域污染源，落实属地行政首长“河长”责任制。新增水质监测断面11个，关闭整治规模畜禽养殖场34家。横荷、东城污水处理厂投入运行，洲心污水处理厂主体工程基本完工，英德望埠、清新污水处理厂加快建设。完成大燕河整治工程和澜水河截污主体工程，龙塘河截污一期工程、笔架河整治工程加快推进。

综合治理大气污染。出台大气污染防治行动方案，开展大气污染综合防治，抓好陶瓷行业二氧化硫、金属拆解行业非法焚烧以及道路、施工扬尘整治，对建筑垃圾和散装物料实施密闭运输。淘汰“黄标车”﹝25﹞1.65万台，实施用电节能改造项目472个，全面完成规模以上水泥熟料生产线脱硝设施建设，淘汰气污染物﹝26﹞项目36个，空气质量在全省排位上升8位。

抓好生态建设和修复工作。大力推进林业重点生态工程建设，完成24.7万亩重点碳汇工程、325.9公里生态景观林带建设。新建成森林公园25个、湿地公园4个、乡村绿化美化示范村204条。抓好“市肺”工程和生态公益林示范区建设。全市森林覆盖率达71.7%，创建成为“省林业生态市”。开展受污染土壤修复试点。整治违法勘查开采矿产资源案件106宗，实施复绿治理矿山17个。创建国家环保模范城市规划通过国家评审。

八、提升基本公共服务和民生保障水平

争创粤东西北首批省教育强市。省教育强县实现全覆盖，成为粤东西北首批省教育强市，高考成绩跃居粤东西北前列﹝27﹞。创建全国义务教育发展基本均衡县﹝28﹞5个，数量位居粤东西北之首。完成村小改造50所，教学点改造167个，新增幼儿园74所。公办学校实现“校校通”全覆盖，“班班通”﹝29﹞覆盖率达到74%。规范管理中小学生校外托管机构。小学、初中公用经费标准分别提高到生均每年950元、1550元，山区和农村边远地区义务教育学校教师岗位津贴标准提高到人均每月700元，为农村学校从教满25年且在农村学校退休的教师发放终身岗位津贴。外来务工人员子女就读公办学校比率达到83.7%，比上年提高14.8个百分点。岭南职业技术学院和碧桂园职业学院建成开学，省级职教基地建设扎实推进。

改造提升基层卫生院。启动20家镇卫生院、4家社区卫生服务中心的升级改造。投入2067万元，为乡镇卫生院配置“五个一”医疗设备﹝30﹞。新增4家平价医院、21个平价诊室，村卫生站和社区卫生服务站全部提供平价药包服务。启动第二批县级公立医院综合改革。首批77名“3+2”培训﹝31﹞学员全部分配到基层医疗机构。与中山大学合作，实施5年培训提升600名基层医师计划。

启动文化体育公共服务均等化建设。出台文化体育基本公共服务均等化实施方案。新建镇（街）综合文化站6个、镇（街）公共电子阅览室15个、村级文化室266个、乡镇农民体育健身工程18个。市县图书馆加快升级改造，市群众艺术馆完成主体工程，市中心图书馆、演艺中心、城市馆（含规划馆与博物馆）、科技馆与青少年宫等市级文化综合体完成建筑方案竞赛。

大力抓好民生实事。建成创业孵化基地13个，组织劳动力技能培训3.3万人，新增城镇就业5.2万人，城镇登记失业率下降到2.36%。全面完成城镇基本养老、医疗、失业、工伤、生育和城乡居保参保扩面任务。6项底线民生保障水平均达到或超过省定标准。保障性安居工程开工3421套，建成3181套。推进10个农贸市场食品安全试点、30家省级食品安全示范学校食堂和2个药品安全示范县创建活动。扎实推进十件民生实事，除部分社区体育公园和国省道大修要延到春节后完成外，其它事项已全部完成﹝32﹞。

过去一年，我们大力推进政府自身建设，扎实开展党的群众路线教育实践活动，重点整治“四风”﹝33﹞突出问题；深入开展“公述民评”活动，查处了一批违法违纪典型案件，解决了一批影响群众切身利益的问题；严格执行中央八项规定，“三公”经费压缩了31%；完善了政府重大行政决策程序，大力推进政务信息公开；更加主动接受人大、政协监督，及时处理群众来信来访，政府形象和社会满意度进一步提升。

此外，我们积极应对禽流感、登革热等疫情，扎实推进各项社会事业发展，国防动员、人民武装、民族宗教、妇女儿童、档案方志等工作都取得了新进步。

各位代表！过去一年政府工作取得的成绩，是在市委的正确领导和统筹谋划下，全市上下开拓进取、真抓实干、奋力拼搏的结果，也是广州、佛山等兄弟市对我们真诚帮助的结果。在此，我代表市人民政府，向所有关心、支持、参与清远建设发展的各界人士，向驻清人民解放军、武警官兵和部省驻清单位，致以衷心的感谢和崇高的敬意！

在梳理工作成效的同时，我们也清醒认识到工作中存在的突出问题：一是，现有产业的质量效益不高，新的主导产业尚未形成，转型发展任重道远。二是，基础设施和产业发展平台还不完善，城市吸引力和承载力有待增强。三是，区域发展仍然不平衡，县域经济发展还面临不少困难和问题。四是，资源环境等要素制约日益突显，保持清远的生态优势面临很大压力。五是，公务员队伍的思想、能力还不适应经济新常态的要求，责任意识和担当意识有所弱化。对此，我们将高度警醒，努力在今后的工作中加以解决。

2015年工作安排

今年是“十二五”规划的收官之年，也是全面深化改革、加快转型发展的关键之年。综合研判，国际国内经济形势总体严峻，经济下行的压力将贯穿全年，资源环境等要素的约束将更加突出，发展面临的困难和挑战不容忽视。但同时，省委、省政府振兴粤东西北将持续发力，广清对口帮扶全方位展开；通过近几年的主动调整，我市大量的基础性工作也已经完成，发展的条件进一步改善；在广清一体化的背景下，我们承接广州产业转移和城市功能外溢的机会前所未有。因此，只要我们把握机遇，保持定力，攻坚克难，扎实推进转型发展，完全可以在新一年取得新的更大进步。

今年政府工作的总体要求是：深入贯彻党的十八大和十八届三中、四中全会以及中央经济工作会议、省委十一届四次全会、市委六届八次全会精神，认真学习领会习近平总书记关于我国经济发展新常态的一系列重要论述，主动适应经济发展新常态，坚持振兴发展、转型发展不动摇；把握稳中求进的工作总基调，狠抓改革攻坚，保持经济回升向好的态势和转方式调结构的定力；坚持分类指导和差异化发展，加大县域发展力度，增加城乡居民收入，继续推进基本公共服务均等化。在全面完成“十二五”规划目标的同时，认真谋划好“十三五”的改革发展任务，为全面建成小康社会奠定基础。

要做好今年的工作，必须突出五个关键：第一，要主动适应经济新常态，彻底摒弃速度纠结，更加注重发展的质量和效益；第二，继续加大投资和产业发展力度，努力保持经济积极向好的态势；第三，高度重视广清协同发展，以大广州思维推动两地全方位对接合作；第四，善用改革思维解决发展中的问题，下更大力气转变政府职能，提升行政效率，激活社会和市场活力；第五，处理好经济发展与民生保障、环境保护的关系，让发展成果最大程度惠及全市人民。

基于以上研判和考虑，建议今年经济社会发展的预期目标和约束指标是：生产总值增长8.5%左右；固定资产投资增长18%左右；地方一般公共预算收入增长9%；社会消费品零售总额增长11%左右；城乡居民收入增长高于地区生产总值的增长；城镇登记失业率控制在3.5%以内，居民消费价格总水平控制在3%左右；城镇化率提高到49.5%左右；社会保障、资源环境保护、节能减排及安全生产等完成省定目标。

围绕实现上述目标，建议今年重点安排八项工作：

一、抓好产业项目的引进和建设

推进一批产业项目落地。提升百嘉科技创新园基础配套，加快广清产业园、华侨工业园、盈富-龙湾工业园、民族工业园以及源潭物流园、华南声谷的开发建设，基本完成顺德清远（英德）经济合作区、英红工业园框架路网建设。通过完善园区配套，力促一批已经签约的产业项目动工建设。以合作招商方式，全面处置闲置、低效用地，缓解项目用地紧张问题。推动项目建设审批流程优化再造，推广项目“直接落地”试点，大幅压缩从立项到施工的平均时间。强化重点项目专人跟踪服务制度，及时解决项目落地涉及到的征地拆迁、民事等问题，完成省、市重点项目年度投资309亿元。

积极对接广州的产业转移。加快广清产业园征地拆迁、路网建设、土规调整、用地报批等工作，完善园区管委会、园区投资开发公司运作机制。争取将广州的产业优惠政策，延伸到广清产业园。积极对接广州商会和行业协会，重点走访有投资意愿的民营企业，引进一批成长性好的中小企业。大力推介清远的区位和成本优势，争取一批服务外包企业落户清远。发挥源潭片区的区位交通优势，重点引进一批税收贡献大的物流企业。争取清远现代化铁路物流项目﹝34﹞尽快落地。

引进和发展新产业新业态。深化与阿里巴巴等电商的战略合作，抓好清远特色馆建设，推进阳山、清新、佛冈、连州农村电商示范县建设；与淘宝大学合作，全面铺开电商创业培训；规划建设电商园区，加强信息网络基础建设，出台加快电商发展优惠政策，完善电商集群注册办法，引进和培育一批电商企业。密切关注城市物流配送、楼宇经济﹝35﹞、汽车服务、服务业超市等新业态的发展走势，争取引进一批新业态示范企业。整合区域旅游资源，规划建设旅游产业集聚区，开展对珠三角旅游投资招商。继续做好金融机构的引进工作。

二、做好城市扩容提质重点工作

加快城市新区基础建设。继续推进燕湖新城14个在建项目建设，力争清晖北路、凤翔大道北、黄腾峡大道、人民东路等在建路网年内建成通车；新开工建设清远大道东段、清晖南路、连石路、沙湖路等新城骨架路网。基本建成北江南岸公园，新开工建设北岸公园和松苏岭公园。完成广清城轨清远站TOD﹝36﹞项目的征地拆迁，并开工建设车站综合体。加快江南水厂一期、太和水厂扩建、220千伏江南站、库清线和110千伏东坑站等供水供电项目建设。加大英德东岸新城征地拆迁力度，力争英德北江三桥建成通车；加快新城起步区排涝泵站排涝水系、安置区首期、横北一号路和滨湖北路等工程建设。完成连州城西新区道路、供水、供电、供气等专项规划和控制性详细规划编制，完成工业大道北段和迎宾大道建设。积极探索PPP﹝37﹞模式，争取将符合要求的项目纳入国家PPP试点。

实施城市改造提升工程。完成中心市区市民体育公园、群众艺术馆等文体设施建设，新建一批社区体育公园，加快笔架河湿地公园和东门塘休闲公园建设，改造提升清新公园和松林公园。规划建设天然气加气站、江南汽车总站及一批公交始末站，新建一批公共停车场，打通老城区一批断头路。编制市区交通信号灯设置规划，统一交通信号灯的建设标准和放行方式。整合平安清远、智能交通和数字城管的信息化建设，打造城市综合管理信息平台和视频监控系统。整治中小学校门口交通秩序，完善指示牌、线标、减速带等设施，配建新建中小学的停车场。推进青山生活垃圾填埋场二期工程和市区建筑垃圾受纳场建设，确保各县（市、区）城市生活垃圾无害化处理设施建成并投入运行，力争市区城市生活垃圾无害化处理率达到90%以上，县城和重点镇达到60%。启动一批城市内涝治理工程。

加大文明城市创建力度。以创建全国文明城市为总抓手，统筹推进国家卫生城市、国家环保模范城市和全国“双拥”模范城市创建活动。把“清远文明蓝丝带”打造成综合性的创文载体和最重要的创文品牌，以关爱、互助为主题，培养城市的人文精神，全面激发市民参与创文的热情。支持各类志愿者组织开展公益性活动，扩大“光盘行动”﹝38﹞、“奇换社区”﹝39﹞、“清远好人”等特色创文品牌影响力。以社区、家庭为单位，深入开展卫生城市创建活动，推进内街小巷、城中村环境卫生整治，在部分住宅小区开展垃圾分类试点，在清城、清新推广使用预拌砂浆。规范门店招牌管理，整合城管、交警、工商、食药监、环保等执法力量，加强城市综合执法管理。

三、构建外联内通的立体交通网络

全面加快对外交通网络建设。推动佛清从高速北段、广清与清连高速连接线工程加快建设；力争汕昆高速龙川至怀集段、汕湛高速清远至云浮段、二广与清连高速连接线上半年全线施工，力争汕湛高速惠州至清远段年内开工；加快连佛高速公路和二广高速连山至贺州支线前期工作。积极对接广州的交通网络，加快广清城际轨道建设，全面打通广清之间的断头路，确保S114线接肇花高速段扩建年内完工，争取原G107线至花都狮岭公路改扩建年内动工。按照广清一体化目标，科学谋划并启动两市新的交通通道建设。结合主干路网建设，加快两市间汽运、物流站场对接。

完成国省道升级改造。加快清连二级公路、银英公路清城段等国省道改造，力争总投资25亿元的33个共633公里“迎国检”公路改造项目上半年完工，实现年内消灭国省道“烂路”、“次差路”目标。加快市区北江四桥、五桥建设。启动321.7公里县乡道烂路改造。继续实行全市“一盘棋”铁腕治超，全面实施国省道路域环境综合整治，推行公路机械化、信息化管养。

铺开北江千吨级航道建设。加快航道建设涉及的征地拆迁、民事协调工作，推进35座丁坝新建修复、31座旧坝拆除等先行工程，力争清远、飞来峡、白石窑三大水利枢纽5座千吨级船闸及坝下33公里航道疏浚工程3月底全面开工。完善航标灯塔等配套设施建设，做好跨河桥梁加固工程。推进北江绿色运输示范项目建设和清远港区规划修编，加快清远港迁建项目前期工作。完成高隆达、安达码头建设，完成正诚、正江、海螺、仁科等码头前期工作。

四、巩固和提升清远的生态优势

提升北江流域治理成效。加大污水处理厂及管网建设力度，确保洲心污水处理厂一期、旧城污水处理厂二期投入使用，完成龙塘河截污一期、连州市区雨污分流管网改造二期工程。开展长湖水库网箱养殖重点整治试点工作。清理“三无”养殖场，完善规模养殖场减排设施。强制报废“四九年船舶”﹝40﹞，完成发证游船的污染排放整治。抓好北江沿线企业监管，确保达标排放。

重拳防治大气污染。优化大气监测站设置，开展大气污染源解析，完成大气预警预报平台建设。继续深化陶瓷、水泥、再生资源等重点行业达标排放治理，加强油气回收治理和燃煤锅炉整治，全面淘汰小锅炉、小冶炼，加快实施餐饮业油烟污染治理。大力整治建设工地及道路运输扬尘，全面实施散体物料密闭运输；密切监控露天采矿和矿石加工场地，实施陶瓷瓷土集中开采配送。积极发展绿色交通，新增公交车辆50%以上使用新能源及清洁燃料，新车登记和外地车辆转入严格执行国四标准。全面推行“黄标车”限行，加快“黄标车”淘汰。确保完成二氧化硫、二氧化氮“十二五”总量减排任务。

加大造林绿化和生态修复力度。严格落实主体功能区生态保护红线，大力推进生态功能区、森林公园建设，积极申报生态公益林示范区建设项目。完成造林38.3万亩、生态景观林带建设280.6公里、中幼林抚育66万亩，新增森林公园和湿地公园13个，绿化美化乡村196条。深化集体林权改革。严厉打击无证开采矿山，抓好矿山复绿。继续推进土壤重金属污染修复工程。扎实开展地质灾害防治，做好地质公园申报建设工作。

五、改善农业农村发展条件

围绕农民增收深化农村改革。继续推动“三个整合”、“三个重心下移”﹝41﹞，提高农村组织化程度，激发农村发展潜力。加强行政引导和政策扶持，鼓励农民在自愿的基础上，组建和参与专业合作社。依托村级公共服务平台和农民专业合作社，大力发展农产品电商销售。整合涉农检测设施，完善网络、村道等基础设施，引导物流向农村延伸，推动电子商务进村入户。以农民专业合作社为单位，开展信用合作社建设，发展农村合作金融。深入推进“政银保”农村金融模式。加大涉农保险推广力度。

实施水利重点工程。加快市区安丰围、龙塘围堤围达标建设，完成15宗列入中央和450公里列入省山区五市中小河流治理项目。抓好高标准基本农田建设，新开工建设32.4万亩。继续抓好省级水利建设示范县、农田水利重点县项目建设，完成一批小型灌区续建配套建设，推进13宗小型水库除险加固项目建设。加快“村村通”自来水工程建设。

完成本轮扶贫开发任务。全力推进扶贫改革试验区建设，完成50个示范村创建，打造一批扶贫改革示范镇。扎实开展扶贫“双到”，确保284个重点帮扶村31118户、97111人稳定脱贫，贫困村集体经济收入全部达到5万元以上。落实新增2751户“两不具备”贫困村移民搬迁安置，完成省农村危房改造任务。积极申报南岭山区“三省五市”集中连片特困区﹝42﹞。做好新一轮扶贫规划。

六、指导支持县域加快发展

切实实行分类指导。继续完善落实差异化配套政策和基本公共服务均等化政策，制订分类考核指标体系，不再考核生态发展区的GDP。帮助北部地区探索生态优势转化为经济优势的有效途径，大力发展绿色农业、生态旅游和增值矿产等产业；推动南部地区加快融入珠三角，主动对接广州等城市的产业转移和功能外溢，提升工业化和城市化水平。推动连阳地区深化区域合作。

加大县域经济发展力度。指导各县（市、区）做好闲置土地清理、城乡建设用地增减挂钩、低丘缓坡开发建设工作。探索城乡建设用地增减挂钩周转指标跨县域流转。充分运用广清帮扶基金，帮助相关县（市、区）解决省市共建高速公路地方资本金筹措问题。完善市、县协同招商机制，推进连阳四县（市）与广州帮扶区共建民族工业园。推动落实少数民族地区基础设施建设免除地方配套政策。帮助生态发展区申报农业项目扶持资金，协调农特产品在广州设立更多销售点。争取尽快启动柳韶铁路建设，力争韶贺高速列入省“十三五”规划。稳定县域人才队伍。

帮助各地解决一批关键问题。赋予高新区、清城区更多的事权，进一步理顺市、区两级责、权、利关系；支持高新区升级为国家级高新区；制定政策支持清城区化解镇（街）历史债务。打通清城、清新之间的断头路，加快清西一级公路建设，加速清新融入大市区。支持沙口神华国华火电项目和黎溪抽水蓄能项目建设，帮助英德加快华侨农场棚户区改造，推动北江大堤英德段达标建设，加快连江流域防洪规划编制工作。争取民族工业园享受省级产业转移园政策和民族优惠政策，协助连州解决“两矿”产业发展中的问题。帮助广清产业园佛冈产业聚集区纳入省重点项目，协助佛冈解决用地规模制约发展问题，争取汕湛高速经行佛冈县城。加快推进连山德建水库建设，积极争取广东瑶族博物馆纳入省级博物馆管理，加快323国道连南、连山县城段改道。支持阳山开展集中连片美丽乡村建设试点，优化矿产资源开发。

七、拓宽民生事业发展渠道

借力广州帮扶加快民生事业发展。推动广州医科大学、广州妇儿中心深化对市人医、市妇幼的帮扶。推动广州医疗机构结对帮扶县级医院，加强县级医院专科建设，提升县级医院综合服务能力。继续推进市县、县镇医疗一体化，完善基层卫生院“五个一”设备配置，提升17家中心卫生院。加快市精神病医院和职业病防治医院建设。整合基层卫生与计生服务资源，推行优生优育，综合治理出生人口性别比，稳定低生育水平。依托广州培训资源，提升清远教师、医生水平。继续推动两地教师交流挂职、跟班学习，建立两市教育教学信息资源共建共享机制。继续推进创建教育强镇活动，实现省教育强镇（乡）全覆盖。启动清城、佛冈、连南创建教育现代化先进县（区）工作。调整中心城镇学校布局，用两年时间基本解决县城和中心镇学校“大班额”问题。启动特殊教育提升计划和新一轮学前教育三年行动计划。加强两市文化体育合作交流，共建市民网络学习中心，提升文化体育场馆建设管理水平。大力开展两市劳务合作。

推动社会办学办医。出台扶持社会办学办医政策措施，放宽准入条件，简化审批程序，加大财政支持力度，优先保障用地。重点扶持建设一批优质民办学校、上等级的综合医院和专科医院。确保广外外校、广铁一中清远分校明年秋季建成开学，力促中山大学医学院美林湖医院尽快落地建设，进一步推动广州名校、名医院到清远办学办医。加快省级职教基地二期工程建设，确保拟入驻的5所高职院校如期动工。

加强民生保障服务。继续实施积极的就业政策，与相关机构合作，实行惠及面更广、更有效的就业培训，力争新增城镇就业5万人，转移劳动力就业4万人。继续扩大社会保障覆盖面，确保企业在岗职工养老保险参保率达90%，缴费率达95%，基本医疗保险参保率稳定在98%以上，实现城乡居民养老保险应保尽保。落实好工人工资支付保证金、欠薪应急周转金和工人工资支付管理政策。实现居家和日间照料服务基本覆盖城市社区和半数以上的农村社区。推进一站式婚姻登记、婚检服务。完善社会救助体系。创建国家级综合减灾示范性社区。

办好十件民生实事。一是提高底线民生保障水平。城乡居民低保补差标准分别提高到374元/月和172元/月；农村五保对象供养标准提高至当地上年度农村居民人均纯收入的60%以上；孤儿基本生活集中供养和分散供养标准分别提高到1240元/月和760元/月；医疗救助人均补助标准提高到1556元/年；残疾人生活津贴和重残护理补贴标准分别提高到1200元/年和1800元/年。二是提高教育经费补助标准。小学和初中公用经费补助标准分别提高到每生1150元/年和1950元/年；残疾学生生均公用经费补助标准提高到小学不低于6000元/年；中职和普通高中国家助学金提高到每生2000元/年；中职学校免学费补助标准提高到每生3000元/年。为全市农村小学及教学点班级提供优质教学资源“班班通”项目。三是为我市户籍35-64岁已婚农村贫困妇女免费提供乳腺癌、宫颈癌检查服务。四是增强城乡劳动力创业就业能力。开展职业技能晋升培训20000人次以上，组织创业培训2500人，扶持创业1000户，带动就业5000人。五是在全市51个农贸市场和较大型超市开展食品安全规范化、标准化建设。六是增设北江流域监测断面，提升水质监测水平。七是全面消灭市域国省道“次差路”，路面改造大修333公里；完成500公里农村公路硬底化建设和100座县乡公路危桥维修加固改造工程。八是新建一批保障性住房。完成保障性住房开工任务4606套（户），基本建成公租房402套，新增低收入住房保障家庭租赁补贴947户。九是整治一批中小河流域。动工15宗中央项目和5宗省试点项目，年内完成工程投资的60%以上；动工一批列入省实施方案的中小河流治理工程，整治河道350公里。十是增加城乡间公共交通站点与班次。完成138个公交候车亭和500个公交站牌建设；市区新投放公交车80辆。

八、提高政府法治化水平

深化政府自身改革。建立完善市县镇村四级综合政务服务体系，实现村居网上办事点全覆盖。出台乡镇行政体制改革方案和配套措施。加强委托、下放、转移事项的监督和业务指导。加快出台第二批政府部门权责清单，清理非行政许可审批事项和行政审批前置项目，推进行政审批标准化建设。加快市县镇村四级公共资源交易平台建设。实行商事登记全程电子化和多证联办，实现商事登记便利化。加强改革调研，完善督办问责机制，推动工作落实。

提升依法治理能力。继续实施重大行政决策公示、专家咨询论证、合法性审查、社会公示与听证会相结合的决策机制。落实市政府常务会议学法制度，充分发挥市政府法律顾问作用，推动建立县级法律顾问制度。落实行政首长出庭应诉工作。加强商事登记后续监管，建立市县一体化的协同监管平台。完善公共财政全口径预算管理，建立公立医院、学校预算管理制度。依法打击各类犯罪活动，全面构建社会治安防控体系。认真落实市人代会及其常委会的各项决定，定期向市人大常委会报告工作。认真接受市政协的民主监督，主动听取各民主党派、工商联、无党派人士和人民团体的意见建议。认真办理人大建议议案、政协提案。

以“两建”提升市民法治意识。继续完善市级信用信息收集管理，启动县级公共信用信息系统建设；推动各类社会主体实施守信公开承诺制度，建立健全失信惩戒社会联动机制和公共信用信息快速披露机制。继续健全商务领域市场主体的信用档案、信用评价和分类管理机制。加强“两建”信息平台后续开发利用，强化部门间信息互通共享，落实企业“黑名单”及经营异常名录管理制度。坚决打击制假售假行为。推进法制宣传，落实普法规划，不断增强市民、企业和社会组织的法治意识。

各位代表，清远的发展正处在关键的节点，全市人民对清远的未来有更多的期待，对美好生活也有更多的向往，我们必须竭尽全力去实现人民的愿望。今年的政府工作任务重、难度大，需要我们加倍努力，务实进取，更有担当。让我们在市委的坚强领导下，坚定信心，迎难而上，积极作为，为建设我们的共同家园不懈努力！
